# Positioning of the dispersive electrode and its effect on the safety and efficacy of radiofrequency ablation

**DOI:** 10.1093/europace/euae296

**Published:** 2024-12-14

**Authors:** Enrique Berjano, Ramiro M Irastorza

**Affiliations:** BioMIT, Electronic Engineering Department, Universitat Politècnica de Valencia, Camino de Vera, Valencia, Valenciana 46018, Spain; BioMIT, Electronic Engineering Department, Universitat Politècnica de Valencia, Camino de Vera, Valencia, Valenciana 46018, Spain; Instituto de Física de Líquidos y Sistemas Biológicos (CONICET), La Plata, Argentina

We have recently read the article entitled ‘Impact of dispersive patch electrode placement on the safety and efficacy of catheter radiofrequency ablation’, a point of view of Futyma *et al*.^1^ The authors state that ‘when the dispersive patch electrodes (DPEs) are placed incorrectly, it can lead to suboptimal lesion formation and potentially increase the risk of adverse events’. However, they do not provide any solid reference to support this claim, and in fact, there is hardly any current evidence on this issue.

In our previous studies based on computational models, we did not observe a significant clinical impact of changing the DPE position on lesion size.^2,3^ Futyma *et al*. cite our two previous works and emphasize some of the limitations of our methodology. However, contrary to what the authors claim, (i) we did compute the lesion depth including extracardiac structures such as lung, connective tissue, and bones and (ii) the conductivity and resistivity of the lungs was taken from a recognized database, which interestingly is the same one used in a recent study based on computer modelling which is indeed well regarded by Futyma *et al*. for presenting results in line with their point of view.

Other limitations of our computer modelling study mentioned by Futyma *et al*., such as the impact of cumulative thermal injury to the oesophagus from multiple sequential and overlapping radiofrequency applications, fluctuations in oesophageal resistivity that can lead to increased current density in the oesophageal region, and the fact that atrioesophageal fistula formation is multifactorial and therefore cannot be adequately modelled by mathematical models, simply have very little to do with the impact of the DPE position on lesion size.

In summary, what we have learned from our previous studies based on physical laws and computer simulations is that the active electrode is actually very far from the dispersive electrode in electrical terms, and therefore, the spatial distribution of current density (causing heating around the ablation electrode) is virtually unaltered by the position of the dispersive electrode. There may be an impact on the baseline impedance caused by the location of the dispersive electrode and the number of patches used, in the same way that body mass index affects the baseline impedance. And this fact will obviously affect the lesion size, since more or less radiofrequency power will be deposited around the active electrode. However, our computational results suggest that there is no redirection of the RF current by repositioning the DPE.

In conclusion, our current view on this point is that there is not enough scientific evidence to claim that repositioning changes the distribution of the radiofrequency current around the active electrode and that repositioning DPE might allow modulating the lesion size simply by varying the baseline impedance, in the same way as changing the programmed power level.
